# Relationship between skill training and skill transfer through the example of bimanual motor learning

**DOI:** 10.1111/ejn.16194

**Published:** 2023-12-11

**Authors:** Marleen J. Schoenfeld, Jude Thom, Jade Williams, Charlotte J. Stagg, Catharina Zich

**Affiliations:** ^1^ Wellcome Centre for Integrative Neuroimaging, FMRIB, Nuffield Department of Clinical Neurosciences University of Oxford UK; ^2^ Medical Research Council Brain Network Dynamics Unit, Nuffield Department of Clinical Neuroscience University of Oxford UK; ^3^ Department of Experimental Psychology University of Oxford UK; ^4^ Medical School University of Oxford UK; ^5^ Department for Clinical and Movement Neurosciences UCL Queen Square Institute of Neurology London UK

**Keywords:** bimanual motor coordination, motor learning, online study, training, transfer

## Abstract

Skill training aims to improve the performance of the task at hand and aims to transfer the acquired skill to related tasks. Both skill training and skill transfer are part of our everyday lives, and essential for survival, and their importance is reflected in years of research. Despite these enormous efforts, however, the complex relationship between skill training and skill transfer is not yet portrayed completely. Building upon two theories, we probed this relationship through the example of bimanual learning with a large cross‐sectional design (N = 450) using an online framework. We designed five training tasks which differed in the variance of the training material (schema theory) and three transfer tasks differing in their similarity to the training task (identical elements theory).

Theoretically, the five training tasks and the three transfer tasks varied approximately linearly from each other. Empirical data, however, suggested merely the presence of three statistically different training tasks and two significantly different transfer tasks, indicating a nonlinear relationship. Against our expectation, Bayesian statistics suggested that the type of skill training was not related to the type of skill transfer. However, the amount of skill training was positively related to the amount of skill transfer.

Together, we showed that motor learning studies can be conducted online. Further, our results shed light on the complex relationship between skill training and skill transfer. Understanding this relationship has wide‐ranging practical implications for the general population, particularly for musicians, athletes and patients recovering from injury.

## Graphical abstract

We probed the relationship between skill training and transfer through the example of bimanual motor learning in an online framework. Five training tasks differing in the variance of the training material (schema theory), and three transfer tasks differing in their similarity to the training task (identical elements theory) were used in 450 individuals. Against our expectation, Bayesian statistics suggested that the type of skill training was not related to the type of skill transfer.

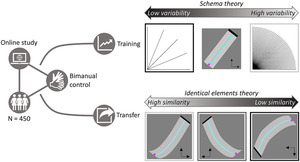

**Abbreviations**
AMI: achievement motivation inventory
Pe: normalized mean error
PI: performance index
Pm: normalized mean movement time

## 1 INTRODUCTION

Almost every aspect of our daily lives involves repeated practice, or training. The objective of training is usually two‐fold: increasing performance in the action at hand, i.e., skill acquisition, and benefitting from the skill when performing other actions, i.e., skill transfer. Across disciplines, the importance of skill transfer has long been known, yet the conditions facilitating skill transfer are still largely unknown. This is not only of theoretical interest but has wide‐ranging practical implications for the general population, in particular for certain groups, such as musicians, athletes and patients recovering from injury. Here we explore the relationship between skill training and skill transfer using a bimanual motor task.

Conceptually, this work builds upon two theories: the schema theory and the identical elements theory. According to the schema theory, a given motor skill is acquired as a generic or especial skill depending on the training (Schmidt, 1975, 2003). In brief, generic motor skills are acquired after variable training when the movement is abstracted to a higher level (Schmidt, 1975, 2003), while especial motor skills are acquired after many repetitions of constant training (Breslin et al., 2010, 2012; Keetch et al., 2005; Schmidt, 1975; Schmidt & Young, 1987; Wulf & Schmidt, 1997). Theoretically, generic motor skills are characterised by larger transfer (Schmidt & Young, 1987; Shea & Kohl, 1990; Shea & Wulf, 2005; Wulf & Schmidt, 1997) than especial motor skills (Keetch et al., 2008, 2005; but see Shea & Kohl, 1991; Shoenfelt et al., 2002).

Secondly, the identical elements theory suggests a positive relationship between the amount of transfer and the similarity between the training and transfer task (Lee, 1988; Magill & Anderson, 2014; Thorndike & Woodworth, 1901). For instance, transfer occurred when the training and transfer task only differed in speed, distance to target and muscles involved (Aune et al., 2017; Keetch et al., 2005; Shea & Kohl, 1990; Simons et al., 2009), but was absent when performing drawing instead of tapping, a new movement sequence or the control‐display relationship was reversed, i.e., transposition (Grafton et al., 2002; Lewis et al., 1951; Robertson et al., 1999; Schoenfeld et al., 2021).

To explore the relationship between training and transfer we use a task probing bimanual motor learning recently published by Schoenfeld et al. (2021). Bimanual motor tasks require the interaction of both hands and have experimentally studied with finger tapping and flexion/extensions tasks (e.g., Aoki et al., 2003; Bangert et al., 2010; Debaere et al., 2004; Kajal et al., 2017; Serrien, 2008), cyclic/rhythmic tasks (e.g., Mueller et al., 2009; Preilowski, 1972; Sisti et al., 2011) and cooperative tasks (e.g., Doost et al., 2017). Across task types, two modes of bimanual interactions are used: equal contribution between hands, e.g., symmetric or mirror movements, or unequal contribution between hands, e.g., asymmetric movements. The task used here was inspired by Doost et al. (2017), who adapted a bimanual version of a circuit game from Lefebvre et al. (2012) to study bimanual motor learning with equal contributions of hands. We believe the task used here is particularly well‐suited for this question as it can be easily adapted in several ways to create sets of training and transfer tasks and it reliably induces learning (Schoenfeld et al., 2021). Motor learning, both bimanual and unimanual, implies motor skill acquisition through repetition and training, which results in lasting improvements in speed and accuracy (Willingham, 1998).

Taken together, we investigate the relationship between training and transfer using an existing bimanual motor task in a web‐based framework. Following the schema theory, we systematically vary the training task covering five levels on an approximately linear scale (from low to high variability), which theoretically ranges from especial to generic motor skill training. Building on the identical elements theory, we use three transfer tasks, which theoretically linearly differ in their similarity to the training task. We hypothesise that more variable training reinforces generic motor skills and thus enables transfer to all three transfer tasks, as reported before (Breslin et al., 2012; Czyż & Moss, 2016; Shea & Kohl, 1990; Wulf & Schmidt, 1997). In contrast, we believe that less variable training reinforces especial motor skills, for which we only expect transfer to tasks that are fairly similar to the training task. Based on the schema theory and the identical elements theory, we hypothesise that transfer is moderated by the variability of training and the similarity between the training and transfer tasks.

## 2 METHODS

### 2.1 Participants and sample size calculation

To avoid any carry‐over effects, we opted for a large cross‐sectional design implemented online. 450 right‐handed individuals (238 females, M = 27 years, SD = 5.96) participated in the study, which was approved by the Central University Research Ethics Committee approval (University of Oxford; MSD‐IDREC‐R61309/RE002) and is in accordance with the Declaration of Helsinki. Consent was given anonymously online.

Data were gathered using Prolific (https://www.prolific.co/; Peer et al., 2017; Palan & Schitter, 2018). Eligibility criteria comprised: age between 18 and 40 years, no neurological or psychiatric disorders, no chronic diseases, no current medication and both hands in good health. Further, eligibility was restricted to fluent English, right‐handedness and a Prolific approval rating above 80%.

The sample size was calculated based on pilot data (independent sample of N = 75) focusing on the interaction between training and transfer task, η^2^_p_ = 0.054. The between‐factor comparison was calculated with an effect size of 0.24 considered to be a medium effect using Cohen's (1988) criteria (>0.26 according to Cohen, 1988, pp. 413–414 and Bakeman, 2005). With a correlation between repeated measures = 0.663, an alpha = 0.05 and power = 0.95, the projected sample size needed with this effect size was N = 420 (calculated with GPower 3.1.9.4).

### 2.2 Experimental design

In the beginning of the experiment, two questionnaires were completed online. Like Schoenfeld et al. (2021), we used a short self‐report questionnaire with scores from 1 (basic) to 3 (expert) to estimate bimanual competence, comprising a range of activities including sports, hobbies, keyboard and phone typing, musical instruments and video games. Additionally, participants completed the short version of the achievement motivation inventory AMI (translated from German ‘Leistungsmotivationsinventar’, Schuler & Prochaska, 2001), a psychological test inventory measuring different aspects of work‐related achievement motivation. Both questionnaires were conducted to determine potential differences across groups.

**FIGURE 1:**
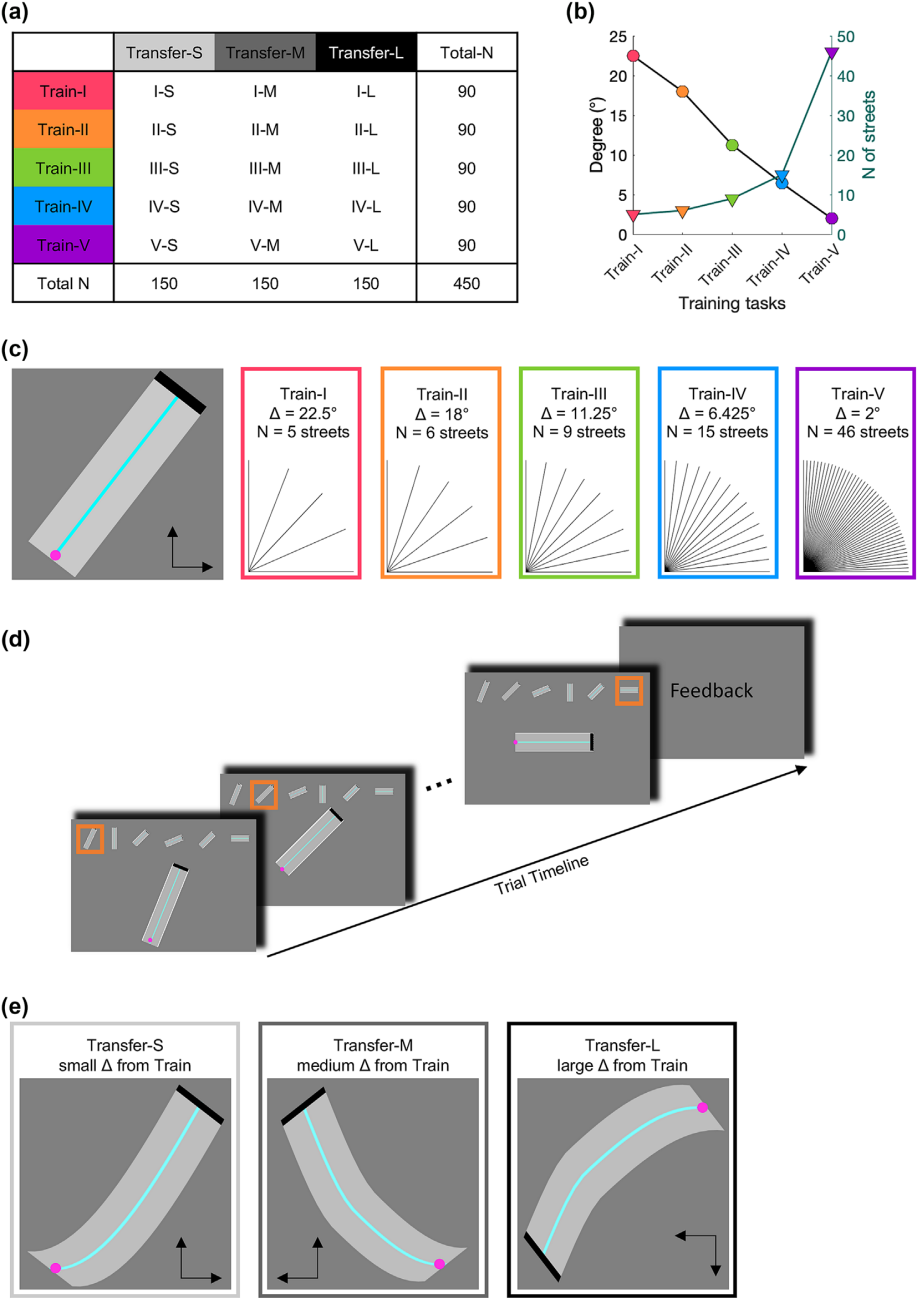
Experimental design and trial structure. (a) Between‐subject design with five training tasks (train‐I, train‐II, train‐III, train‐IV, train‐V) and three transfer tasks (transfer‐S, transfer‐M, transfer‐L) resulting in 15 individual groups (each N = 30). (b) The five training tasks varied in their angular difference between streets (black line and circles), which results in differences in the number of streets (green line and triangles). (c) One street from the training task (left) and all street angles for each training task. (d) Trial structure. One trial consisted of a path of six streets. The whole path was displayed at the top of the screen, whereby the current street, shown enlarged in the centre, was highlighted in orange. At the start of each street, the cursor was at the starting position. When the cursor reached the end of the street, the next street started immediately. If the cursor hit either side of the street, the cursor was reset to the starting position. Feedback about movement time and accuracy was provided at the end of each trial and the inter‐trial interval was self‐paced. (e) All transfer tasks utilised curved streets. The three transfer tasks differed in their similarity, expressed as the direction of cursor movement (indicated by the arrows), to the training task.

After the questionnaires were completed, the task was performed. We used a between‐subject design with five training tasks (Train‐I, Train‐II, Train‐III, Train‐IV, Train‐V) and three transfer tasks (Transfer‐S, Transfer‐M, Transfer‐L), resulting in 15 individual groups (each N = 30, Figure 1a). Data were acquired group‐wise; group order was pseudo‐randomised. Each participant completed questionnaires and three task sections: one of three transfer tasks (pre), one of five training tasks and the same transfer task again (post), whereby one street for familiarisation, was performed before each section.

Across all training and transfer tasks, the aim was to navigate a cursor on a street as fast and accurately as possible. All training tasks comprised only straight streets angled between 0° and 90°, whereby the street angles reflected the level of bimanual control. Specifically, only one hand was required for the angles 0° and 90°, both hands were required equally for the angle of 45° and all other angles corresponded to more complex interactions between the two hands. The five training tasks differed in their minimum angular difference between streets (Train‐I: 22.5°, Train‐II: 18°, Train‐III: 11.25°, Train‐VI: 6.425°, Train‐V: 2°). Thus, the angular difference between neighbouring training tasks was on average 5.13° with a standard error of 0.55°, approximating a linear relationship across training tasks. Differences in the minimum angular difference across the five training tasks resulted in a different number of streets across the five training tasks (Figure 1b, c). For example, Train‐I comprised five different streets with street angles of 0°, 22.5°, 45°, 67.5° and 90°, while Train‐V comprised 46 different streets (0°, 2°, 4°, … 90°). The order of streets was pseudo‐randomized, such that all streets within one training task were repeated equally, and with a minimum of 22.5° between concurrent streets to avoid street‐to‐street carry‐over effects.

All five training tasks were performed for 100 trials (see Schoenfeld et al., 2021 for a discussion on a number of repetitions versus time of training), thus the actual time on task was comparable across the five training tasks. Each trial equalled one path comprising six streets (Figure 1b). At the beginning of each street, the cursor was at the starting position (i.e., in the middle of the width of the path, at the bottom‐left position). The cursor could be moved upwards and to the right using the two keys. If the cursor hit either side of the street, the cursor was reset to the starting position. When the cursor reached the end of the street (defined as the last 5% of the whole length of the street) the next street started immediately. After each trial, the trials' movement time and error (see section 2.5 Statistical analysis for more details) was fed back to the participant. To minimise fatigue and distraction the inter‐trial interval was self‐paced (Rowe et al., 2002). On average the inter‐trial interval was 6.6 s (*SE* = 0.17 s) across subjects and trials.

All transfer tasks comprised only curved streets (Figure 1d, see Supplemental Figure S1). The transfer task was performed for 5 trials before and 5 trials after training. Our previous work showed that 5 trials are sufficient to evaluate transfer effects, especially in large sample sizes. More trials can yield practice‐related changes within the transfer task. Each trial comprised six slightly different curves, whereby their order was pseudo‐randomized. The trial structure was identical to the training tasks.

In contrast to straight streets, navigating the cursor on curved streets required constant adaptation of bimanual control. The three transfer tasks differed in their similarity to the training tasks, which were implemented by changing the direction of cursor movement. Thus, for Transfer‐S cursor direction was the same as in the training tasks, i.e., upwards and to the right. Transfer‐M had one cursor direction transposed, i.e., upwards and to the left, wherefore the streets from Transfer‐S were flipped along the vertical axis. Finally, Transfer‐L had both cursor directions transposed, i.e., downwards and to the left, wherefore the streets from Transfer‐S were flipped along both the vertical and horizontal axis. The only difference between neighbouring levels (i.e., Transfer‐S and Transfer‐M; Transfer‐M and Transfer‐L) was the flip of one dimension. Therefore, we hypothesised that the difference between Transfer‐S and Transfer‐M, and the difference between Transfer‐M and Transfer‐L were comparable and linear.

### 2.3 Online data acquisition

Online studies provide the ideal framework to reach a large and diverse sample. Proof‐of‐concept and reliability reports suggest high conformity between data acquired web‐ and laboratory‐based (Anwyl‐Irvine et al., 2021; Bridges et al., 2020). The task was implemented using PsychoPy3 v2020.1.3 Builder GUI. Running PsychoPy experiments online requires HTML and JavaScript files, which were automatically generated using PsychoJS. Therefore, the task was exported to PsychoJS v2020.1 and uploaded to Pavlovia (www.pavlovia.org), an online server hosting the study. Participants accessed the task via their web browser.

The use of participants' devices was restricted to desktop computers or laptops, though their operating systems varied (i.e., Windows: N = 320, Mac: N = 118, Linux: N = 12). The use of a Chrome browser was recommended due to its high temporal accuracy (Anwyl‐Irvine et al., 2021) and the screen refresh rate was set to 60 Hz. To account for variable screen sizes across participants we used height units, which scale stimuli relative to the height of the participants' window (Peirce, 2021).

Participants' keyboards served as response devices, whereby a standard QWERTY/QWERTZ keyboard was recommended. The two keys, ‘K' and ‘S', enabled horizontal and vertical cursor movement, respectively. The keys were used with an acceleration rate of 0.001 height units (i.e., when holding a key, the cursor would accelerate 1/1000 height units of the study window per refresh rate). Therefore, velocity was relative to the duration a key was pressed and would potentiate. Maximum velocity was set to 0.3 height units. In turn, if the keys were released, the cursor decreased its velocity gradually in the same way.

### 2.4 Data pre‐processing

Across all training tasks, street angles ranged from 0° to 90°, with street angles reflecting the level of bimanual control. Therefore, some streets and thus trials were easier than others. To avoid confounding learning‐related changes with trial difficulty, data were denoised. Denoising was conducted for the two dependent variables, movement time and error, separately. First, street difficulty was quantified by averaging the dependent variable for each street angle across all street occurrences (i.e., within and across subjects for each training task separately). Street angles of 0° and 90° were easiest, while street angles between 10° to 40° and 50° to 80° were most difficult (Supplemental Figure 2). The trial difficulty was simply the average of the street difficulties of the corresponding street. Next, a linear regression was fitted with trial difficulty and the group‐level performance in movement time or error for each training task separately. The raw residuals depicted the trial difficulty in each training task and were subtracted from single‐subject data (Supplemental Figure 3). The advantages of this denoising were no data loss while preserving single subject variance.

### 2.5 Dependent variables

To quantify performance, error and movement time were combined into a performance index (PI; Fleming et al., 2017; Lefebvre et al., 2012, 2013). To this end, movement time and error were obtained per street. Movement time was defined as the time from cursor movement onset to offset. Error was defined as the root mean square of the physical distance between the cursor position and the ideal line (using a sampling rate of 60 Hz). The distance was obtained with Heron's Formula for straight streets and with the nearest neighbour using Pythagoras theorem for curved streets. From pilot data (not shown), the error and movement time of the 75 subjects (5 subjects per group) were averaged to obtain constant error ‘a’ and constant speed ‘b’ values. Normalized mean error (Pe = a/trial‐wise error) and normalized mean movement time (Pm = trial‐wise movement time/b) were used to compute the PI (PI=Pm*Pe). Trial‐wise performance was obtained by averaging across the six streets per trial.

### 2.6 Statistical analysis

To investigate changes in the learning task, the PI of the first (early) and last (late) ten trials of the training task were compared. To investigate changes in the transfer task pre and post‐PI transfer were compared. To examine differences in change across learning tasks, change scores (i.e., the difference between early and late PI of the training tasks) were calculated. Similarly, to examine differences in change across the transfer tasks, change scores (i.e., difference between pre and post‐PI of the transfer tasks) were calculated.

If not stated otherwise, Frequentist statistics were used. Data were analysed using paired samples t‐test and two‐sample t‐test, analysis of variance (ANOVA) and analysis of covariance (ANCOVA). In case of significant effects, post hoc t‐tests were conducted with Bonferroni correction for multiple comparisons. Correlations were assessed using Pearson's correlation. All reported p‐values for t‐tests were two‐tailed and the significance level was set at *p* < 0.05.

The main interaction, i.e., time x empirical training task and empirical transfer task (see section 3.4) was followed up using Bayesian statistics to assess if the data favoured the null hypothesis compared to the alternative hypothesis (Dienes, 2014). To this end, we conducted the same analysis as above (van den Bergh et al., 2020; van den Bergh et al., 2022) using default priors and report Bayes Factor exclusion (BF_exclusion_) and for the interaction effect. Statistical analyses were performed using the open‐source software JASP (version 0.16.4, JASP Team, 2022).

## 3 RESULTS

### 3.1 Empirical data confirmed different levels of training and transfer tasks

**FIGURE 2:**
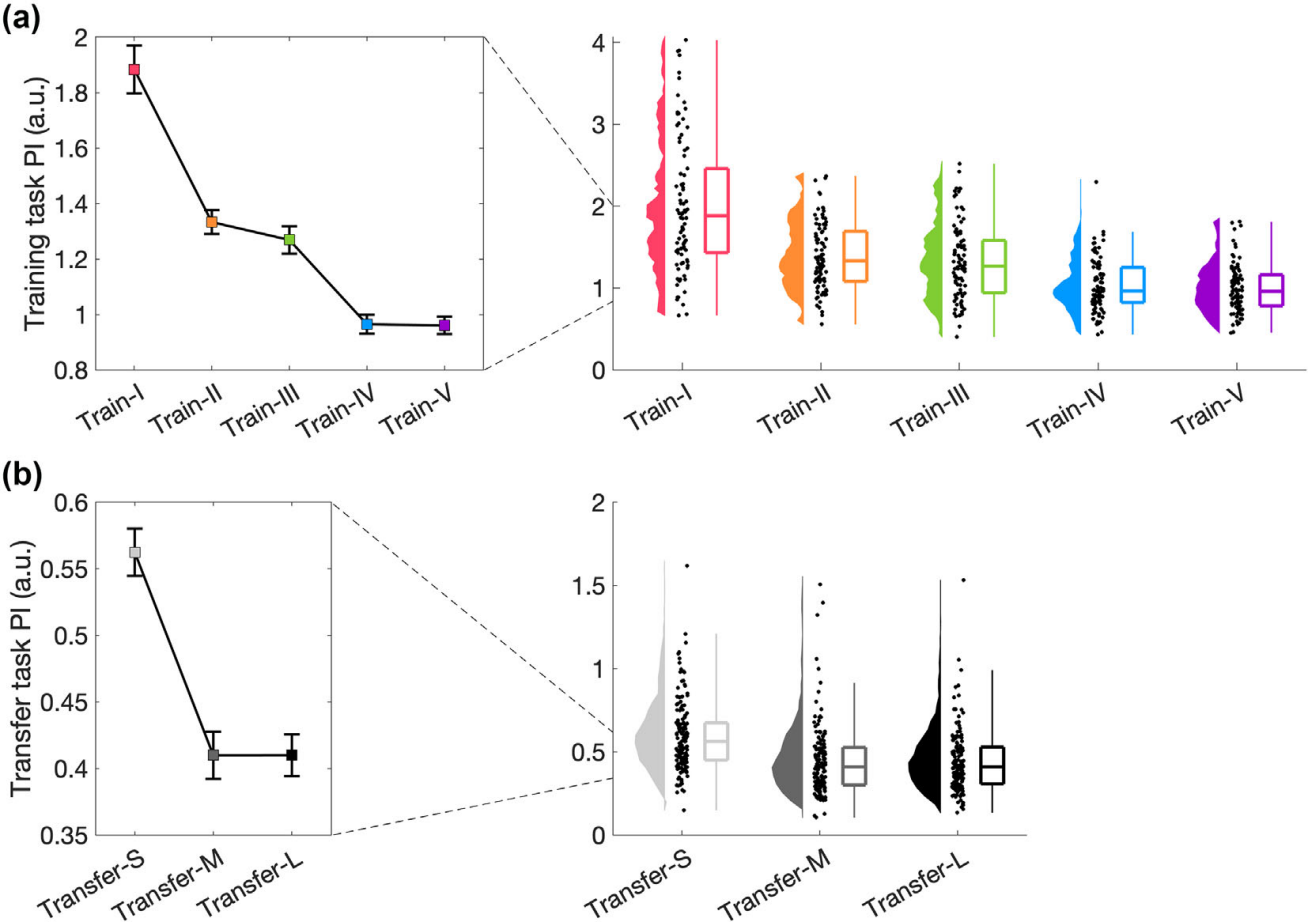
Empirical validation of training and transfer tasks. (a) Validation of training tasks. Performance was averaged across all trials (N = 100) for each of the five training tasks (N = 90). Median and standard error across subjects is shown (left) as well as single subject data, distribution, and boxplot (right). (b) Validation of transfer tasks. Performance was averaged across post‐trials (N = 5) for each of the three training tasks (N = 150). Median and standard error across subjects is shown (left) as well as single subject data, distribution, and boxplot (right).

First, we empirically validated the training and transfer tasks. This was crucial, as different levels of training and transfer tasks constituted a prerequisite for our main analysis, i.e., whether different training tasks differentially affected skill transfer. Therefore, we asked whether the five training tasks differed from each other using a 1 × 5 ANOVA with the between‐subject factor *training task* (Train‐I to Train‐V) and the dependent variable performance, i.e., across all trials. We found a significant main effect of the training task (F_4,445_ = 59.506, *p* < 0.001, η^2^_p_ = 0.348, Figure 2a) and all but two (Train‐II vs Train‐III and Train‐IV vs Train‐V) follow‐up t‐tests were significant (Table 1). This suggested that empirically not five, but three levels (Train‐I, Train‐II/III, Train‐IV/V) could be distinguished. The three empirical levels differed as follows: Train‐I vs Train‐II/III: t_268_ = 8.438, *p* < 0.001, Cohen's *d* = 1.086; Train‐I vs Train‐IV/V: t_268_ = 14.360, *p* < 0.001, Cohen's *d* = 1.859; Train‐II/III vs Train‐IV/V: t_358_ = 8.702, *p* < 0.001, Cohen's *d* = 0.915.

**TABLE 1:** Post‐hoc t‐test of 1 × 5 ANOVA with the between‐subject factor training.

		Mean difference	SE	t	Cohen's d	*p*_bonf_
Train‐I	Train‐II	0.619	0.075	8.298	0.955	< 0.001***
	Train‐III	0.674	0.075	9.034	1.009	< 0.001***
	Train‐IV	0.979	0.075	13.116	1.569	< 0.001***
	Train‐V	1.011	0.075	13.558	1.638	< 0.001***
Train‐II	Train‐III	0.055	0.075	0.736	0.125	1.000
	Train‐IV	0.359	0.075	4.818	0.975	< 0.001***
	Train‐V	0.392	0.075	5.260	1.097	< 0.001***
Train‐III	Train‐IV	0.305	0.075	4.083	0.758	< 0.001***
	Train‐V	0.338	0.075	4.524	0.862	< 0.001***
Train‐IV	Train‐V	0.033	0.075	0.442	0.106	1.000

*p*‐value adjusted for comparing a family of 5. Cohen's d does not correct for multiple comparisons.

Next, we aimed to validate our transfer tasks. As the transfer tasks were designed to differ in their similarity to the training tasks, we performed one 1 × 3 ANCOVA with the between‐subject factor *transfer task* (Transfer‐S, Transfer‐M, Transfer‐L), the dependent variable performance, i.e., post‐transfer, and pre‐transfer performance as covariate. A significant main effect of transfer task (F_2,446_ = 19.741, *p* < 0.001, η^2^_p_ = 0.081, Figure 2b) was followed up with t‐tests, which revealed that Transfer‐S differed significantly from Transfer‐M (t_446_ = 3.722, *p <* 0.001, Cohen's *d* = 0.340) and Transfer‐L (t_446_ = 6.252, *p <* 0.001, Cohen's *d* = 0.591), but Transfer‐M and Transfer‐L did not differ significantly from each other (t_446_ = 2.138, *p* = 0.081, Cohen's *d* = 0.193). Therefore, although theoretically, the transfer tasks had three levels, empirically only two levels could be observed (Transfer‐S, Transfer‐M/L). The two empirical levels differed as follows: Transfer‐S and Transfer‐M/L: t_448_ = 7.326, *p* < 0.001, Cohen's *d* = 0.731.

Together, analysis of our empirical data demonstrated a 3 (Train‐I, Train‐II/III, Train‐IV/V) × 2 (Transfer‐S, Transfer‐M/L), rather than a 5 (Train‐I to Train‐V) × 3 (Transfer‐S, Transfer‐M, Transfer‐L) design. On this basis, Train‐II/III; Train‐IV/V; as well as Transfer‐M/L were combined for subsequent analyses.

### 3.2 Questionnaires

For both questionnaires, we performed a 3 × 2 ANOVA with the between‐subjects factors *empirical training task* (Train‐I, Train‐II/III, Train‐IV/V) and *empirical transfer task* (Train‐S, Train‐M/L), and the dependent variable questionnaire score. For bimanual motor competence, we found a significant main effect of *training task* (*F*_2,443_ = 8.316, *p* < 0.001, η^2^_p_ = 0.036). Follow‐up t‐tests revealed that individuals in Train‐I had less bimanual competence than individuals in Train‐II/III (t_443_ = −4.075, *p <* 0.001, Cohen's *d* = −0.553) and Train IV/V (t_443_ = −2.590, *p =* 0.030, Cohen's *d* = −0.355). The main effect of *transfer task* as well as the t*raining task x transfer task* interaction were not significant (both *p's* > 0.1). For achievement motivation, we performed the same analysis, however, no significant effects were observed (all *p's* > 0.1).

### 3.3 Performance improved across all training tasks

**FIGURE 3:**
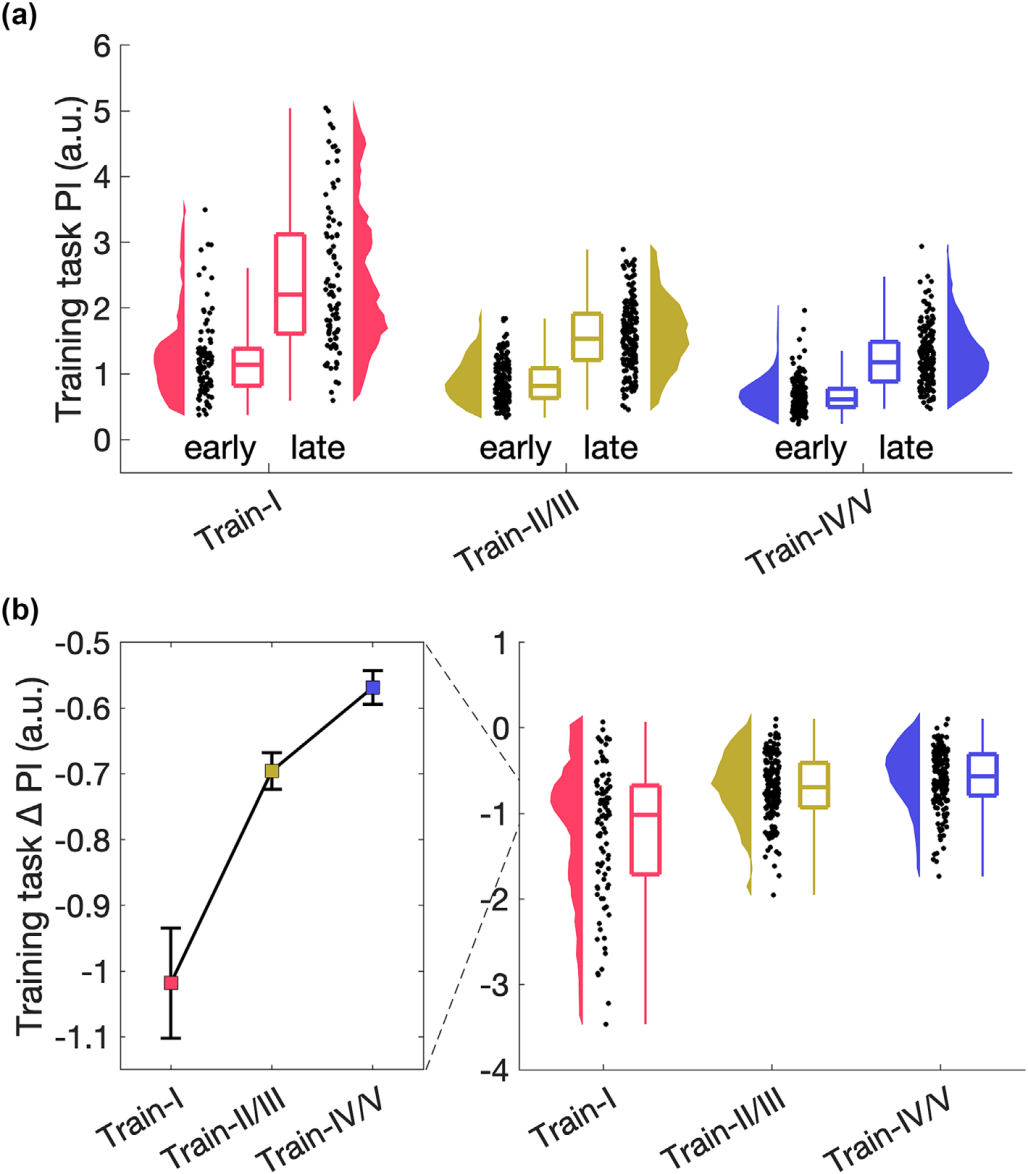
Training‐related changes for the three empirically different training tasks. (a) Training‐related changes for the three empirically different training tasks (N = 90; 180; 180, respectively) with performance averaged across the first (early) and last (late) ten trials. Data are shown on single subject level, distribution, and boxplot. (b) Change in performance, i.e., difference between the first and last ten trials. Median and standard error across subjects is shown (left) as well as single subject data, distribution, and boxplot (right).

As skill learning was a prerequisite to assess skill transfer, next we evaluated whether learning‐related changes were induced. Paired samples t‐tests, comparing the first and last ten trials, confirmed improved performance for all training tasks after practice (Figure 3a, Train‐I: *t*_1,89_ = −14.457, *p* < 0.001, Cohen's *d* = −1.524; Train‐II/III: *t*_1,179_ = −25.177, *p* < 0.001, Cohen's *d* = −1.877; Train‐IV/V: *t*_1,179_ = −22.867, *p* < 0.001, Cohen's *d* = −1.704). To investigate whether training‐related effects differed across the three empirical training tasks and whether bimanual competence influenced learning, we performed a 3 × 3 ANOVA with the between‐subjects factors *empirical training task* (Train‐I, Train‐II/III, Train‐IV/V) and *bimanual competence* (basic, medium, expert), with the dependent variable performance change, i.e., change between the first and last ten trials. A significant main effect of *training task* was observed (*F*_2,440_ = 45.542, *p <* 0.001, η^2^_p_ = 0.172, Figure 3b). Follow‐up t‐tests revealed that learning was higher in Train‐I compared to Train‐II/III (*t*_440_ = −7.880, *p <* 0.001, Cohen's *d* = −1.128) and Train‐IV/V (*t*_440_ = −9.124, *p <* 0.001, Cohen's *d* = −1.287). Furthermore, we found a significant main effect of *bimanual competence* (*F*_2,440_ = 5.697, *p = 0*.004, η^2^_p_ = 0.025), and follow‐up t‐tests revealed that individuals with basic bimanual competence learned less than individuals with medium (*t*_440_ = 3.319, *p =* 0.003, Cohen's *d* = 0.453) and expert bimanual competence (*t*_440_ = 2.656, *p =* 0.025, Cohen's *d* = 0.400, **Supplemental** Figure 4). There was no significant *empirical training task* x *bimanual competence* interaction (*p >* 0.1).

### 3.4 Relationship between skill training and skill transfer in light of the schema theory and the identical elements theory

Our results suggested three distinct training tasks and two distinct transfer tasks. Moreover, results demonstrated significant changes over the course of practice, demonstrating skill training. We hypothesised that the skill transfer would be modulated by the type of skill training. To test this, a 2 × 3 × 2 ANCOVA was performed with a within‐subject factor of *time* (pre and post) and between‐subject factors of *empirical training task* (Train‐I, Train‐II/Train/III, Train‐IV/Train‐V) and *empirical transfer task* (Transfer‐S, Transfer‐M/L) with transfer task performance as dependent variable, and *learning‐related change* as well as *bimanual competence* as covariates.

**FIGURE 4:**
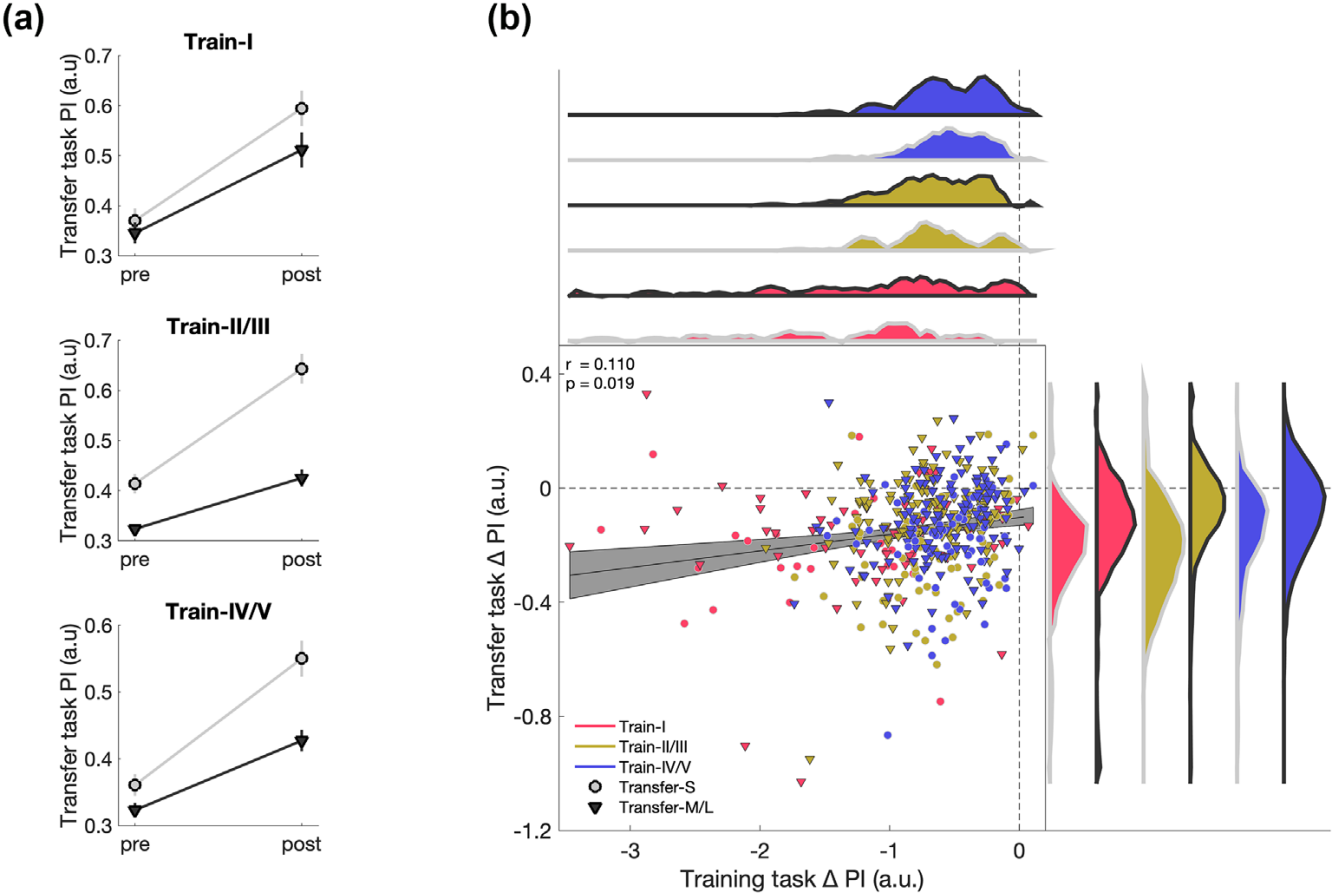
Relationship between training tasks and transfer tasks. (a) Non‐significant time x empirical training task x empirical transfer task interaction. Performance of the transfer task is shown for the two empirical transfer levels (i.e., transfer‐S, grey circles; transfer‐M/L, black triangles) for pre and post as well as for each of the three empirical training tasks (i.e., top: train‐I, middle: train‐II/III, bottom: train‐IV/V). Mean and standard error of the mean are displayed. (b) Correlation between change in training task and change in transfer task. Grey area represents the 95% confidence intervals. Distributions for each subgroup are colour‐coded, respectively.

The significant main effect of *transfer task* (F_1,441_ = 33.702, *p <* 0.001, η^2^_p_ = 0.071) mirrored the effect reported in section 3.1, i.e., Transfer‐S and Transfer‐M/L differ significantly from each other. The significant main effect of *time* (F_1,441_ = 9.652, *p =* 0.002, η^2^_p_ = 0.021) demonstrated that performance was better in post‐transfer compared to pre‐transfer. The significant covariates *training‐related change* (F_1,441_ = 18.916, *p <* 0.001, η^2^_p_ = 0.041) and *bimanual competence* (F_1,441_ = 10.446, *p =* 0.001, η^2^_p_ = 0.023) indicate that higher performance change during the training task and higher bimanual competence yield better performance in the transfer task. Further, we observed a significant *time* x *training‐related change* interaction (F_1,441_ = 8.315, *p =* 0.004, η^2^_p_ = 0.019), indicating a relationship between the amount of training‐related change and amount of transfer. There was no significant interaction between time x covariate: *bimanual competence* (*p* > 0.1). Against our expectations, no significant interactions between the training task and the transfer task were found (all *p*'s > 0.1). The *time* x *empirical training task* x *empirical transfer task* interaction is visualised in Figure 4a. This main interaction was followed up using Bayesian statistics to assess if the data favoured the null hypothesis compared to the alternative hypothesis (Dienes, 2014). Results suggested substantial evidence (BF_exclusion_ = 5.644) for the null hypothesis (i.e., there is no interaction between factors) compared to the alternative hypothesis (i.e., there is an interaction between factors). Together, these results demonstrated that skill transfer was not affected by the different types of training tasks used here. However, the amount of skill transfer was related to the amount of skill learning. To investigate this further, we performed a correlation analysis between the change in the training task and the change in the transfer task across all subjects, which revealed a positive correlation (*r* = 0.110, *p =* 0.019, Figure 4b).

## 4 DISCUSSION

Through the example of bimanual motor control, we probed the relationship between skill training and skill transfer. While we used bimanual motor control here as a showcase, the results will likely extend to other domains and will be useful for providing practical guidelines for skill acquisition in general. Building upon the schema theory and the identical elements theory, we quantified the amount of transfer from a set of five training tasks (from low to high variability; schema theory) to a set of three transfer tasks (from small to large difference to the training task; identical elements theory). The large 5 × 3 between‐subject design comprising 450 individuals was conducted using a web‐based framework. Our data suggested a discrepancy between theoretical and empirical task difficulty, indicating that the difficulty of bimanual motor tasks was not mapped linearly in humans. Further, against our expectations, Bayesian statistics provided substantial evidence against an interaction between the type of skill training and skill transfer. However, we found that the amount of skill learning positively correlated with the amount of skill transfer, independent of the training and transfer tasks. Together, our data showed that motor learning studies can be performed online and that the relationship between skill training and skill transfer cannot be solely explained by the schema theory and the identical elements theory.

### 4.1 Discrepancy between theoretical and empirical training task levels

Based on the schema theory, a motor skill is acquired as a generic skill after variable training, or a especial skill after many repetitions of constant training (Breslin et al., 2010, 2012; Keetch et al., 2005; Schmidt, 1975; Schmidt & Young, 1987; Wulf & Schmidt, 1997). Building on this theoretical framework, we used a set of five training tasks (Train‐I, Train‐II, Train‐III, Train‐IV, Train‐V), which differed in the minimum angular difference on an approximately linear scale to map the range from constant to variable training.

Results showed that empirically (i.e., based on the average performance) three training tasks, rather than five, could be distinguished. Specifically, Train‐I showed better performance than Train‐II/III, which in turn showed better performance than Train‐IV/V. This observed discrepancy between theoretical and empirical task levels, indicates that the levels of bimanual motor control may be more complex and not mapped linearly in humans.

### 4.2 Discrepancy between theoretical and empirical transfer task levels

To investigate the relationship between skill training and skill transfer, we used three transfer tasks (Transfer‐S, Transfer‐M, Transfer‐L).

The transfer tasks were inspired by our earlier work (Schoenfeld et al., 2021) as well as the identical elements theory. In brief, in Schoenfeld et al. (2021) 5 differently angled streets with an angular difference of 22.5° between streets were practised for a minimum of 100 trials. The transfer was assessed on the same streets, whereby the cursor‐hand configuration was swapped. No transfer was observed. We believe no transfer was observed because the training was not variable enough for the fairly different transfer task. In other words, to observe transfer the training task should have been more variable, or the transfer task should have been more similar to the training task.

To quantitatively map this training‐transfer‐relationship, we used several training and transfer tasks. While the training tasks differed in the variability of the training, the transfer tasks differed in their similarity to the training task. Specifically, Transfer‐S used curved streets and the same movement direction as the training task (i.e., left to right and bottom to top). Transfer‐M and Transfer‐L utilised the same streets, but the streets were mirrored along one or both axes requiring a different movement direction (Transfer‐M: right to left and bottom to top; Transfer‐L: right to left and top to bottom). We believe that these transfer tasks were not confounded by the effect of proximity (Wrisberg et al., 1987). One could argue that the three transfer tasks did not only differ in their similarity to the training task but also in their difficulty. However, without any prior task knowledge, the three transfer conditions should be comparable. Transfer‐S only appears to be less difficult than Transfer‐M and Transfer‐L after the training task, because Transfer‐S and the training task were conducted using the same movement direction, or in other words because Transfer‐S was more similar to the training task than Transfer‐M and Transfer‐L. If the training task had used the same movement direction as Transfer‐L, Transfer‐L would appear easier than Transfer‐S and Transfer‐M, because Transfer‐L would be more similar to the training task. In order words, in the framework of this study, the difficulty of the transfer task is a function of the similarity between training and transfer task.

Results showed that empirically (i.e., based on the post‐performance) not three, but two training tasks could be distinguished. Specifically, Transfer‐S showed better performance post‐training, accounting for the pre‐training performance, than Transfer‐M and Transfer‐L. The empirical similarity between Transfer‐M and Transfer‐L might be because changing the movement direction of one hand interfered with the learned movement direction of the other hand. Thus, similar to Transfer‐L, the control between the two hands had to be re‐established under the new rules.

### 4.3 Relationship between skill acquisition and skill transfer

To test the relationship between skill acquisition and skill transfer, we examined performance in three empirically different training tasks and two empirically different transfer tasks. Based on the schema theory and the identical elements theory, we expected that training with high variability (i.e., Train‐IV/V) would induce transfer to all transfer tasks and that training with low variability, i.e., constant training, (i.e., Train‐I) would only transfer to transfer task with a small difference to the training task. However, we did not find evidence for this relationship between training task and transfer task. Indeed, using Bayesian statistics, we found evidence against this relationship.

This is in line with other studies failing to show the advantage of variable training (advantage for constant training: King & Newell, 2013; Shea & Kohl, 1990, 1991; no difference between constant and variable training: Kerr & Booth, 1978; Moxley, 1979; Mattar & Ostry, 2007; Shoenfelt et al., 2002). Regarding force, less error (Shea & Kohl, 1991) and greater changes in the time‐ and frequency‐dependent properties (King & Newell, 2013) were observed for constant, compared to variable, training. The benefits of constant practice were underlined by Keetch et al. (2005), arguing that with training the recall schema becomes more refined. According to the authors, this is why especial skills, i.e., those that emerge after constant practice, have an advantage over the actions from other movements of the same class (Breslin et al., 2010, 2012; Keetch et al., 2005). Overall, these findings supported the viewpoint of specificity of practice (Henry, 1968; Proteau, 1992; Tulving & Thomson, 1973), stating that training conditions should closely reflect those of the transfer conditions. Similar patterns were observed in other domains including mental imagery (Coelho et al., 2012), speech‐motor learning (Rochet‐Capellan et al., 2012) and gait dynamics (Rhea et al., 2012).

However, this contrasts with the variability of the practice hypothesis derived from the schema theory stating that task variation is important for the development of schemata (Braun et al., 2009; Schmidt, 1975; Wulf & Schmidt, 1997). Wulf and Schmidt (1997) found greater transfer effects following variable training compared to constant training, whereby the transfer was similar to the training task, suggesting the presence of a proximity effect (Wrisberg et al., 1987). Using a series of studies, Braun and colleagues concluded that for randomly varying tasks of the same structure, the motor system could extract the task structure and thereby exhibit structure‐specific facilitation, interference reduction and exploration (Braun et al., 2009).

The heterogeneous pattern of results is consistent with a critical analysis of the schema theory by Van Rossum (1990). 73 experiments (from 1975 to 1987) were analysed to evaluate the empirical basis of the variability of the practice hypothesis. For adults, only 12 experiments met the criteria, of which only two experiments clearly supported the variability prediction, six reported limited, weak or partial support and four rejected the prediction (Van Rossum, 1990). Thus, empirical evidence for the schema theory is not as clear as one might have expected, whereby this meta‐analysis merits an update.

Our results further confirmed previous results showing a relationship between the amount of training‐related change and the amount of transfer (e.g., Aune et al., 2017; Mattar & Ostry, 2007). While this relationship was independent of the specific training or transfer task, we found a significantly larger training‐related change in Train‐I compared to Train‐II/III and Train‐IV/V. Note that the participants in Train‐I also showed the least bimanual competence, indicating more potential for training‐related change.

### 4.4 Practical relevance and experimental considerations

Generalisation of any acquired skill to structurally similar skills or other contexts is crucial for human behaviour. For motor abilities, skill transfer appears to be seamless in daily life. For example, learning to catch and throw a ball at a young age is easily transferred to throwing other objects, to other targets, over varying distances and across the lifespan. One could argue that transferability in daily life is promoted, amongst others, by the natural variability in everyday life. Thus, one might naturally transfer learning because the conditions under which a ball is thrown (such as the size of the ball, the target distance and height and the environment) are never identical. On the other hand, improving motor skills in the context of sport and rehabilitation builds much more on structured repetitive practice, often under very similar conditions. Therefore, it is crucial to identify principles that facilitate skill transfer and actively incorporate these into effective training and rehabilitation regimes. Regarding sports, it has been shown that the transfer of motor, conceptual and perceptual variables occurs when sports are similar (Gorman et al., 2011). For example, soccer and rugby, as well as other invasion sports, demand perceptual elements of tracking the ball in flight (Causer & Ford, 2014).

Despite this central role of skill transfer, it is not yet possible to reliably quantify skill transfer. For experimental studies, a major challenge constitutes the choice of the transfer task, which is a balancing act. Simply put, transfer tasks should be different enough from the training task to avoid proximity effects while assessing the key feature(s) of the acquired skill. If the training and transfer tasks are too similar, ‘transfer effects' might be observed, but interpretations are likely confounded by the effects of proximity (Van Rossum, 1990). If, on the other hand, the training and transfer tasks are too different, ‘transfer effects' might be absent as different features were assessed (e.g., Schoenfeld et al., 2021). Lee et al. (1985) provided an interesting framework utilising one transfer goal that was within (or ‘inside’) the range of features varied during the skill acquisition and one transfer goal that was not within (or ‘outside’) of the limits experienced during skill acquisition. This division might have paved the way to assess skill transfer using more than one transfer task. In one of the few studies investigating more than one transfer task, Mattar and Ostry (2007) showed that with increasing difference between training and transfer task, the extent of transfer was reduced. This was confirmed by Aune et al. (2017) in a study using three transfer tasks, which differed in two dimensions (lateral, bilateral & homologous, non‐homologous). The authors found that transfer was lowest in the transfer task that differed most from the training task (i.e., bilateral non‐homologous transfer). Thus, we believe utilising multiple transfer tasks to map different levels of similarity between the training and transfer task is recommendable. Here, we used three transfer tasks, and although Transfer‐L was thought to be more dissimilar from the training task than Transfer‐M, empirical data did not show a difference between the two transfer tasks. This further underlined the challenge of quantifying transfer and designing transfer tasks.

### 4.5 Feasibility of online studies in the area of motor control

The number of online studies has grown enormously in the past few years. Web‐based studies uniquely enable researchers to reach a large, diverse sample in a cost‐ and time‐efficient manner. The majority of online studies are conducted in the area of social and cognitive science, while motor control is lagging behind. It has been suggested that the reduced experimental control may be particularly problematic for movement‐related data. However, the first paper has demonstrated a close correspondence between lab‐ and web‐based results (Tsay et al., 2021). This was further underlined by studies systematically testing for differences between laboratory and online studies indicating high comparability in terms of precision and accuracy of timings for visual and auditory stimuli or response times (Anwyl‐Irvine et al., 2021; Bridges et al., 2020). To ensure high‐quality data from web‐based experiments experiment creation, hosting and recruitment are critical (see Sauter et al., 2020 for a detailed overview). Here we used Prolific as a recruitment platform due to its transparency, usability and multiple pre‐screening options (Palan & Schitter, 2018). In addition, a study by Peer et al. (2017) showed that Prolific's participants are more honest and internationally diverse than other recruitment platforms. However, online experiments share certain limitations, i.e., the experimenter is unable to control the participants environment, which might be noisy and thus distracting. The bimanual motor task used here was previously used in two studies (N = 40, N = 54) in a lab‐based setting using force grippers (Schoenfeld et al., 2021). Across the three studies (two lab‐based and one web‐based), we observed good data quality, the same relationship between performance and bimanual motor control as well as comparable learning‐related change. Therefore, we believe that our bimanual motor task was successfully translated from a lab‐ to a web‐based framework.

## AUTHOR CONTRIBUTIONS

**Marleen J. Schoenfeld:** Conceptualisation; methodology; investigation; formal analysis; writing—original draft. **Jude Thom:** Conceptualisation; writing—review and editing. **Jade Williams:** Analysis; writing—review and editing. **Charlotte J. Stagg:** Conceptualisation; methodology; resources; funding acquisition; supervision; writing—review and editing. **Catharina Zich:** Conceptualisation; methodology; supervision; formal analysis; writing—review and editing.

## CONFLICT OF INTEREST STATEMENT

The authors declare that the research was conducted in the absence of any commercial or financial relationships that could be construed as a potential conflict of interest.

### PEER REVIEW

The peer review history for this article is available at https://www.webofscience.com/api/gateway/wos/peer-review/10.1111/ejn.16194.

## Supporting information


**Figure S1.** Curved streets used in Transfer tasks. (**A**) Curved streets were developed with a curvature of 40°, 50° and 55°. The resulting three curved streets were rotated at 180° to obtain six streets for each trial. These streets were used for Transfer‐S. (**B**) Same as in (A), but streets were flipped along the vertical axis. These streets were used for Transfer‐M. (**C**) Same as in (A), but streets were flipped along the vertical and horizontal axis. These streets were used for Transfer‐L.
**Figure S2.** Relationship between street angle and performance indicated relationship between street angle and street difficulty. (**A**) Movement time for each street angle averaged within and across subjects for each training task separately. Training tasks are highlighted by different colours and circle size. (**B**) Same as (A), but for error.
**Figure S3.** Denoising on exemplary data for one training task (Train IV). (**A**) Movement time before (brown) and after (petrol) denoising. Average and standard error across subjects (N = 90) is shown. (B) Residuals of the fitted linear regression that were subtracted out of single‐subject data. (C) Same as A, but for error. (D) Same as B, but for error.
**Figure S4.** Main effect of training task and bimanual competence.

## Data Availability

The raw data is available at https://data.mrc.ox.ac.uk/bimanual-motor-task and has DOI: 10.5287/ora‐jne9aa1z0. The code for the experimental paradigm is available at: https://github.com/cathazi/bimanual-motor-online-task and the analysis code is available at: https://github.com/cathazi/bimanual-motor-online-analysis.
