# Supplementary material for: Relationship between skill training and skill transfer through the example of bimanual motor learning
Source: Eur J Neurosci. Author manuscript; Available in PMC 2024 Feb 29. (PMC7615689; doi:10.1111/ejn.16194)
Supplement: Supplemental Information [file EMS194265-supplement-Supplemental_Information.pdf]

Supplemental Information

Relationship between skill training and skill transfer through the example of bimanual motor learning

Marleen J. Schoenfeld, Jude Thom, Jade Williams, Charlotte J. Stagg, Catharina Zich

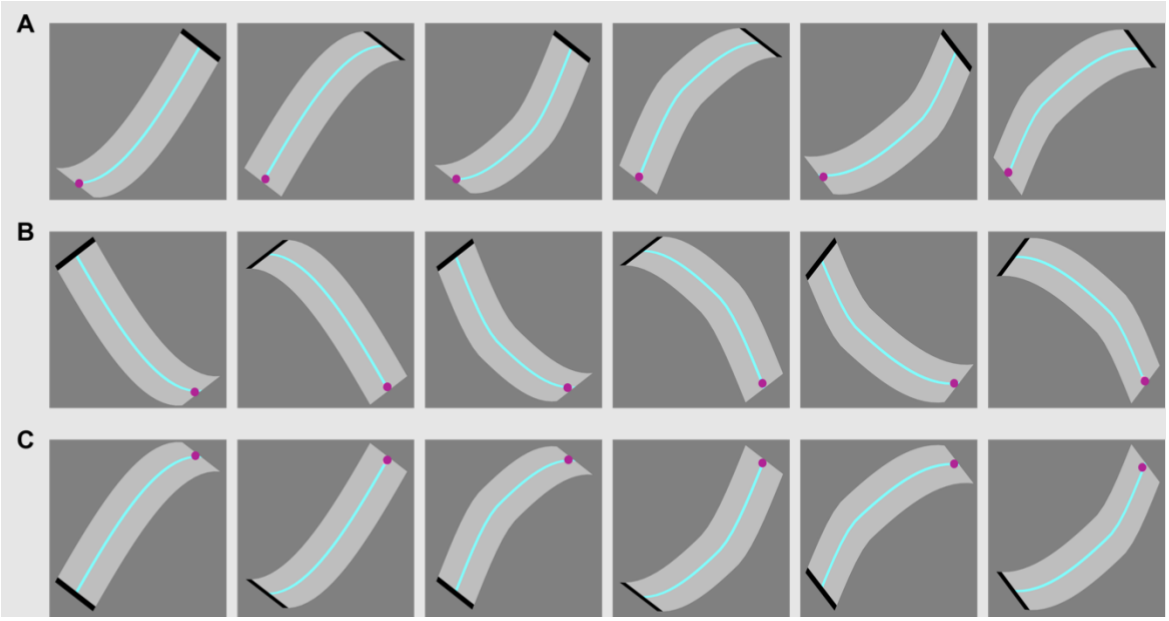

Supplemental Figure 1. *Curved streets used in Transfer tasks.*
*(**A**) Curved streets were developed with a curvature of 40°, 50° and 55°. The resulting three curved streets were rotated at 180° to obtain six streets for each trial. These streets were used for Transfer-S.*
*(**B**) Same as in (A), but streets were flipped along the vertical axis. These streets were used for Transfer-M.*
*(**C**) Same as in (A), but streets were flipped along the vertical and horizontal axis. These streets were used for Transfer-L.*

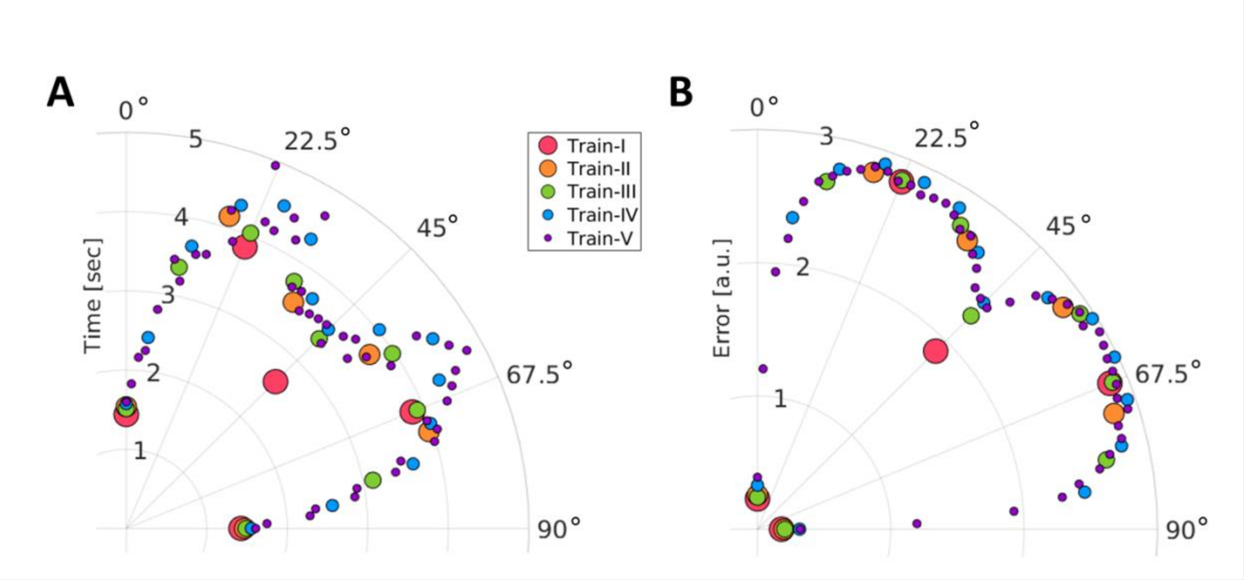

Supplemental Figure 2. *Relationship between street angle and performance indicated relationship between street angle and street difficulty.*
*(**A**) Movement time for each street angle averaged within and across subjects for each training task separately. Training tasks are highlighted by different colour and circle size.*
*(**B**) Same as (A), but for error.*

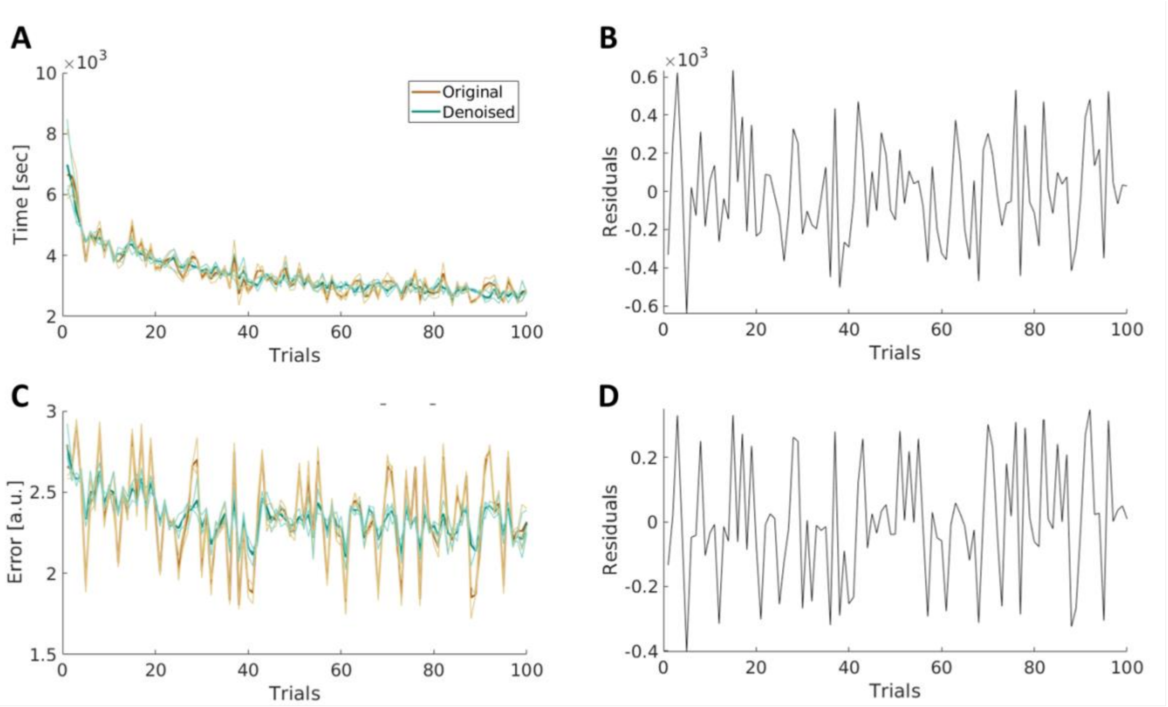

***Supplemental Figure 3**. Denoising on exemplary data for one training task (Train IV).*
*(**A**) Movement time before (brown) and after (petrol) denoising. Average and standard error across subjects (N = 90) is shown.*
*(**B**) Residuals of the fitted linear regression that were subtracted out of single subject data.*
*(**C**) Same as A, but for error.*
*(**D**) Same as B, but for error.*

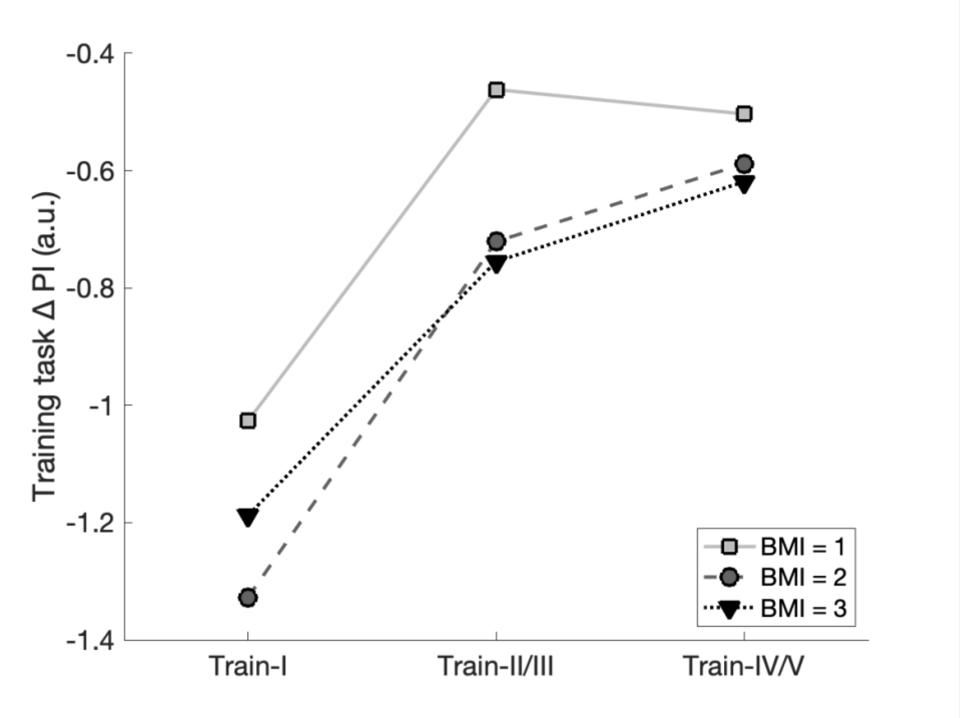

Supplemental Figure 4. *Main effect of training task and bimanual competence.*
